# dbPTM in 2019: exploring disease association and cross-talk of post-translational modifications

**DOI:** 10.1093/nar/gky1074

**Published:** 2018-11-10

**Authors:** Kai-Yao Huang, Tzong-Yi Lee, Hui-Ju Kao, Chen-Tse Ma, Chao-Chun Lee, Tsai-Hsuan Lin, Wen-Chi Chang, Hsien-Da Huang

**Affiliations:** 1 Warshel Institute for Computational Biology, The Chinese University of Hong Kong, Shenzhen 518172, China; 2 School of Science and Engineering, The Chinese University of Hong Kong, Shenzhen 518172, China; 3 School of Life and Health Science, The Chinese University of Hong Kong, Shenzhen 518172, China; 4 Department of Computer Science and Engineering, Yuan Ze University, Taoyuan 32003, Taiwan; 5 Institute of Tropical Plant Sciences, College of Biosciences and Biotechnology, National Cheng Kung University, Tainan 70101, Taiwan

## Abstract

The dbPTM (http://dbPTM.mbc.nctu.edu.tw/) has been maintained for over 10 years with the aim to provide functional and structural analyses for post-translational modifications (PTMs). In this update, dbPTM not only integrates more experimentally validated PTMs from available databases and through manual curation of literature but also provides PTM-disease associations based on non-synonymous single nucleotide polymorphisms (nsSNPs). The high-throughput deep sequencing technology has led to a surge in the data generated through analysis of association between SNPs and diseases, both in terms of growth amount and scope. This update thus integrated disease-associated nsSNPs from dbSNP based on genome-wide association studies. The PTM substrate sites located at a specified distance in terms of the amino acids encoded from nsSNPs were deemed to have an association with the involved diseases. In recent years, increasing evidence for crosstalk between PTMs has been reported. Although mass spectrometry-based proteomics has substantially improved our knowledge about substrate site specificity of single PTMs, the fact that the crosstalk of combinatorial PTMs may act in concert with the regulation of protein function and activity is neglected. Because of the relatively limited information about concurrent frequency and functional relevance of PTM crosstalk, in this update, the PTM sites neighboring other PTM sites in a specified window length were subjected to motif discovery and functional enrichment analysis. This update highlights the current challenges in PTM crosstalk investigation and breaks the bottleneck of how proteomics may contribute to understanding PTM codes, revealing the next level of data complexity and proteomic limitation in prospective PTM research.

## INTRODUCTION

Protein post-translational modifications (PTMs) are one of the most important mechanisms of eukaryotic and prokaryotic cells, which involve the attachment of chemical groups to amino acid side chains of proteins (1,2). A variety of PTMs have been reported to play crucial roles in diversely cellular processes that modulate a protein’s function, physicochemical properties, conformation, stability and molecular interactions in response to developmental signals or environmental stimuli (3,4). For instance, protein phosphorylation is the most ubiquitous PTM that induces signal transduction and cell apoptosis (5); protein acetylation and methylation are involved in chromatin reprogramming and transcriptional regulation (6,7); lysine glutarylation plays a crucial role in metabolic pathways and mitochondrial functions (8); lysine ubiquitination mediates protein degradation (9); protein glycosylation is in charge of controlling the cell-extracellular matrix interactions (10,11). In the last decade, high-throughput mass spectrometry (MS) has enabled studies to identify a large-scale modified proteomes (10). Detecting PTMs with the use of MS or MS/MS-based proteomics is a rapidly developing technology, which provides practical means for the site-specific identification of modified peptides (12). Up till now, over 200 different types of PTMs have been discovered through MS/MS-based proteomics (13); a couple of resources (14–17) have thus been developed for accumulating multiple PTM types with functional annotations. The difficulty of integrating heterogeneous datasets from a variety of PTM databases has inspired the expansion of dbPTM (18) to be a comprehensive resource by systematically retrieving experimentally verified PTMs from public domains and research articles.

In the past few years, an increasing number of studies have reported that the dysregulation of PTMs is implicated in the development and progression of several diseases, including cancer (19–22). Song *et al.* identified that parkin may inhibit cell growth by decreasing ribosomal protein SA (RPSA) expression and inducing phosphorylation of cytokeratin 8/18 (23). Kang *et al.* also discovered that the increased O-GlcNAcylation in non-small cell lung carcinoma A549 cells can respond to glucose deprivation and block adenosine triphosphate synthesis, which may play a functional role in the survival of cancer cells (24). Although MS/MS-based proteomics has substantially improved our knowledge of the substrate-site specificity of various PTM types, there is an emerging demand to explore the functional roles of PTMs on proteins, especially for the investigation of disease-associated PTMs. Multiple PTM substrate sites within a single protein can collectively modulate a biological outcome (25). The crosstalk of combinatorial PTMs has been reported to be involved in a majority of cellular processes (26). Extensive molecular studies of well-known PTMs such as phosphorylation and O-linked glycosylation have validated the premise that phosphorylation for activation of the Akt-TSC2-MTOR signaling cascade is derived by O-GlcNAcylation from UDP-N-acetylglucosamine, which can reveal novel functional roles of fructose in promoting embryonic growth and development during pregnancy (27). Additionally, Wu *et al.* have unraveled a molecular mechanism underlying sumoylation-regulated p53 function and further uncovered a new role of acetylation in antagonizing the inhibitory effect of sumoylation on p53 binding to DNA (28). Although recent studies have provided new insights into the interplay between different PTMs, research into the frequency and functional relevance of PTM crosstalk would be worthwhile to clarify potential roles of modified proteins in regulatory mechanisms of cells.

Advanced MS-based technology has not only brought a surge in proteome-scale studies, but also contributed to a fruitful list of identified PTMs. However, with the increase in the number of identified PTMs, the most crucial question is what kind of disease these PTMs are involved in. High-throughput deep sequencing has led a genome-wide analysis of association between single nucleotide polymorphisms (SNPs) and diseases into a data surge in both growth and scope. Single amino-acid polymorphisms (SAPs) are amino acid variations corresponding to the genetic variation of non-synonymous SNPs (nsSNPs) (29). The amino acid variants may result in functional changes of corresponding proteins, such as changes in active sites and functional domains, which is the type of variation most frequently related to human diseases (30). The disease-associated nsSNPs that alter PTM sites can be utilized to estimate the various PTM candidates involved in other diseases (31). This is particularly potent in light of the fact that most protein-based pharmaceuticals deliver their therapeutic effects through some form of SNPs. Despite this, our understanding is still limited with respect to the local effects and frequency of SAPs near PTM substrate sites. Therefore, an integrated analysis between SNPs, PTMs and diseases is necessary to understand the role of PTMs in the development and progression of diseases. This update highlights the current state of SNP–PTM association study and breaks the bottleneck of how genomics may contribute to understanding PTM-disease relationship. Owing to the relatively limited information regarding the frequency and functional relevance of PTM crosstalk, this update has developed an automatic system to detect the PTM sites neighboring with other PTM sites in a specified window length, subjected to motif discovery and functional enrichment analysis (FEA). These contemporary analyses not only conduct a thorough investigation of disease-associated PTM site but also showcase a higher level of data complexity and prospects for disease-related PTM studies.

## IMPROVEMENTS

In this update, the database not only integrates more experimentally validated PTMs from publicly available databases and through manual curation of the literature but also provides PTM crosstalk analysis and PTM-disease associations. The highlighted improvements and advances in the dbPTM 2019 update are displayed in Supplementary Figure S1, including (i) update of the experimental PTM dataset from published databases and literature, (ii) update of the benchmark datasets for PTM analyses, (iii) update of the existing PTM-related databases and tools, (iv) investigation of PTM-disease associations based on single amino acid polymorphisms (SAPs), (v) investigation of PTM crosstalk between two different modification types and (vi) improvement of the user interface. The details of each process are described below.

### Data update for experimentally verified PTM substrate sites

MS/MS-based proteomics has inspired the development of an increasing number of databases for accumulating experimentally validated data of various PTM types. In this update, a systematic pipeline is adopted to gather experimentally verified PTM sites from 30 available PTM-related resources. Supplementary Table S1 presents the statistics of the experimental and putative PTM sites from all the integrated PTM databases. In addition to database integration, a pipelined text extraction system (32) has been applied to retrieve research articles with the site-specific identification of modified peptides. Extracting findings from PTM-related literature can enable a full understanding of PTM functions. Nevertheless, the surge in scope and scale of research articles has proved a remarkable challenge in the verification of data correctness. Thus, after the retrieval of PTM-related articles published between 2016 and 2018, we manually curated modified peptides from the published literature. Then, all the collected PTM peptides were mapped to UniProtKB (13) protein entries, and the precise positions of the PTM substrate sites determined based on sequence identity.

This update has integrated a significantly increased number of experimental PTM sites compared to the previous version of dbPTM. To provide further functional and structural information about PTM substrates, dbPTM has been expanded to a knowledgebase comprising not only basic information of protein and PTM substrate sites annotations, but also SAPs, drug and disease associations, and their supporting literature. The functional and structural annotations relevant to the modified proteins and the PTM substrate sites have been updated in this version. Supplementary Table S2 compares the amount of data for each integrated resource between this update and the previous version of dbPTM.

### Update of benchmark datasets for PTM analyses

Tandem mass spectrometry, also known as MS/MS, is a bottom-up proteomic approach that provides a wealth of information about dynamic changes in the modified state of proteins. However, identifying the substrate specificity by using MS/MS-based experiments is labor-intensive, time-consuming and technically challenging. Consequently, a variety of computational methods (33–36) have been proposed to identify putative PTM sites based on protein sequence and structural information. Although most of the studies claimed that their methods could provide better predictive performance than previous studies, there is no standard dataset developed for a fair comparison of the predictive powers among the various PTM prediction tools. Due to the lack of the consistent testing data, it is hard to determine which prediction tool has the best prediction ability solely based on their own cross-validation performances. Hence, this update has compiled non-homologous benchmark datasets for 30 PTM types in an attempt to provide a unified evaluation of predictive performance for various PTM prediction tools. With reference to the established benchmark dataset described in previous work (18), after eliminating the homologous peptides, the statistics of the benchmark datasets for different PTM types is presented in Supplementary Table S3. Based on these benchmark datasets, the test results can offer a suggestion to analysts who need to predict PTM sites with high sensitivity, high specificity or balanced accuracy.

### Expansion of PTM analysis resource portal

With the rapidly growing experimentally-verified PTM sites from MS/MS-based proteomic techniques, more and more online databases and tools have been developed for PTM analyses, including PTM data warehousing, computational prediction of PTM sites, structural investigation of PTM substrate sites and reconstruction of PTM regulatory networks (3,5,37–39). However, given a protein, it is usually difficult to comprehensively analyze PTM functions based on a variety of online databases or tools. Thus, one of the aims in this update was to provide a fully functional and structural investigation of different PTM types present on the proteins of interest. Therefore, the dbPTM has been redesigned to be an integrated resource portal to allow for comprehensive PTM analyses. Supplementary Table S4 lists the number of integrated PTM databases, the database’s name, the number of integrated PTM tools and the tool’s name for each PTM type. The web interface of the integrated resource portal has been enhanced to allow users to access these integrated databases and tools efficiently.

### Data integration for disease associations of PTM substrate sites based on SAPs

**Figure 1.**
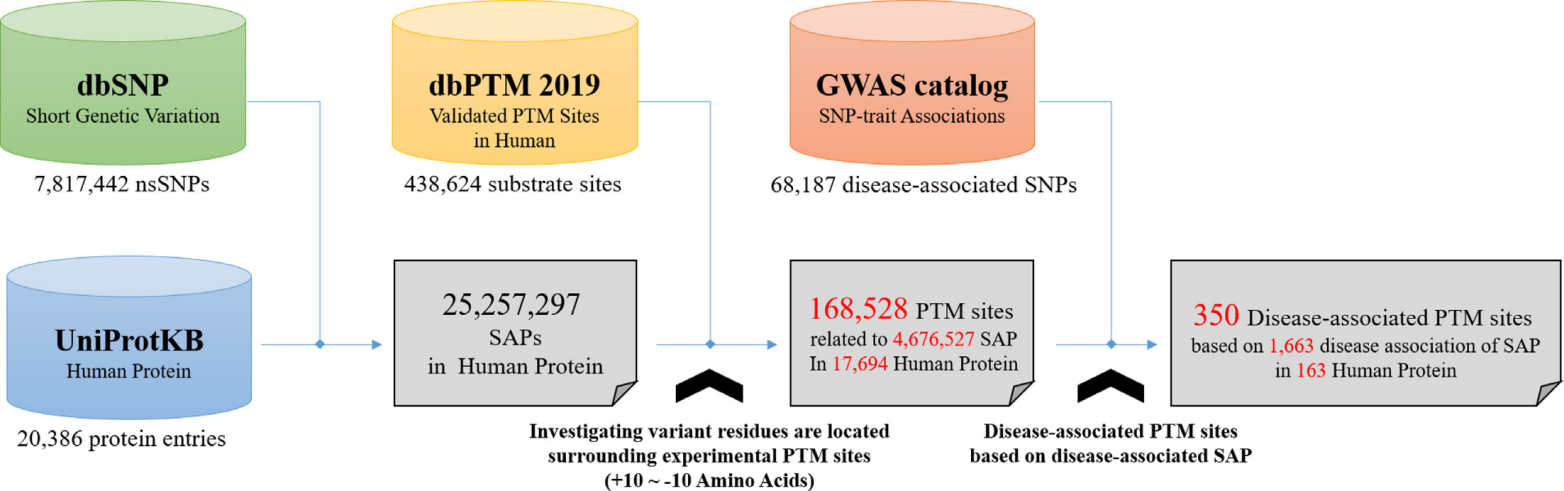
The process of investigating disease-associated PTM sites based on disease association of SAP.

Several studies have provided new insights into the association between PTM substrate sites and nearby disease-related SAPs (29,40–42). This update thus integrates disease-associated SAPs from dbSNPs (43) based on genome-wide association studies (GWAS) (44). The PTM substrate sites encoded within a specified distance from nsSNPs are referred to as having an association with the involved diseases. As shown in Figure**1**, owing to the limited information of protein variants annotated in UniProtKB, all the SAPs are obtained from dbSNP and are mapped to UniProtKB protein entries to determine the precise positions of residue change on a full-length protein sequence. In summary, there are 4,676,527 variant residues locating in the neighboring regions, starting from upstream 10 amino acids to downstream 10 amino acids, of 168,528 PTM sites. In this investigation, the PTM sites, which have disease-related amino acid variations in their neighboring regions, are regarded as disease-associated PTM sites. After large-scale screening of all experimentally verified PTM substrate sites, a total of 350 disease-associated PTM sites containing 1,663 disease-associated SNPs were identified based on the disease annotation of dbSNP.

### Investigation of crosstalk between two different PTM types

In recent years, increasing evidence for crosstalk between PTMs has been discovered (45–49). Although MS/MS-based proteomics has substantially improved our knowledge about the substrate-site specificity of a specific PTM, there is no existing method dedicated to characterizing the crosstalk of combinatorial PTMs that may act as the modulator for protein function and activity. Due to the relatively limited information about the frequency and functional relevance of PTM crosstalk, we performed a systematic process to extract all experimentally-verified PTM sites with additional PTM sites located in a specified window length (−10 ∼ +10AA). The PTM sites within a specified distance of each other were regarded as co-occurring and having the potential for PTM crosstalk between the substrate proteins. In this investigation, a total of 169 PTM pairs were subjected to the motif discovery of two different PTM types and FEA of the substrate proteins. As depicted in Supplementary Figure S2, this update includes an interactive platform for users to analyze proteins containing multiple PTM types. Given a PTM type of interest, the platform produces a table displaying all possible pairs of two PTM types with the number of substrate sites of other PTM types in the relative location of the interested PTM sites. When a user selects a PTM pair, clicking on the number buttons can display the motif of interplay between the two PTM types. The total amount of substrates that are concurrently modified by the PTM pair is also shown in the first column. The results of FEA, including biological processes, molecular function, cellular components and metabolic pathways, are graphically visualized. A negative value of the logarithm of *P* values (−*log_2_P*) is adopted to represent the significance of functional categories, according to the *t*-test against the occurrences of function terms associated with a specific PTM pair (e.g. O-glycosylation and phosphorylation). The significance score for each functional term is colored in blue, with the corresponding significance level displayed in a table.

### Enhancement of the dbPTM web interface

The dbPTM has provided a user-friendly interface for biologists to investigate the functional and structural analyses of PTMs in detail. However, improvements were still required to enhance the ease of accessing the modified proteins and their PTM sites of interest. In particular, we now provide three different ways to let users access a specific type of PTM: ‘Browse by substrate protein’, ‘Browse by summary table’, and ‘Browse by PTM type’. The advanced search function has also been introduced to efficiently reach the PTM data that meet the querying criteria. Moreover, we provide comprehensive relevant information about PTMs, including graphical visualization of PTM sites with structural characteristics and functional domains, a table of experimental PTM sites with the original article, orthologous conservation of PTM substrate sites, disease-associated PTM sites based on SAPs, protein–protein interactions and domain–domain interactions, table of drug and disease associations and literature related to PTMs. It is worth mentioning that ‘Exploring disease-associated PTMs based on SAP’ and ‘Investigation of PTM crosstalk between two different types’ are two newly developed functions in this update, which can be accessed via the ‘Analysis’ item of the navigation menu at the top of web page. Additionally, a summary table of co-occurrences of PTMs (crosstalk analysis) has been established for eight types of PTM, including acetylation, methylation, N-linked glycosylation, O-linked glycosylation, phosphorylation, S-nitrosylation, succinylation and ubiquitination.

## DATA CONTENT AND UTILITY

### Data statistics of PTM sites in dbPTM 2019

The previous version of the database contained a total of 610,037 experimentally-verified PTM sites from 14 external PTM-related databases. To provide the most comprehensive information about PTM substrate sites, this update has accumulated many MS/MS-identified PTM peptides from external data resources and by manually curating articles. It should be highlighted that a total of 30 PTM databases are integrated into this update. After removing the redundant data among these heterogeneous resources, a total of 908,917 experimentally verified PTM sites are obtained from 92,648 research articles. When compared to the previous version, the number of experimentally validated PTM sites has increased by nearly 300,000 in this update. The number of putative PTM sites also has been increased to 347,984. Additionally, Table 1 gives the statistics of experimental and putative substrate sites for each PTM type. Protein phosphorylation, which is the most popular research topic in proteomics community, contains the most abundant data of experimentally verified substrate sites. A total of 571,032 experimentally verified phosphorylation sites were extracted from 52,381 research articles. The next most abundant PTMs are protein acetylation and ubiquitination, which consist of 137,442 and 118,495 experimentally verified substrate sites, respectively. In addition to integrating more experimental data for the previously existing PTM types, the dbPTM has been expanded to accumulate the modification sites for more than 130 PTM types.

**Table 1.** Data statistics of experimental and putative PTM sites for each PTM type in dbPTM 2019

PTM type	Number of experimental sites	Number of putative sites	Number of literatures
Phosphorylation	571032	121330	52381
Acetylation	137442	28811	21251
Ubiquitination	118495	34950	1130
Succinylation	17596	5184	62
Methylation	17483	18036	8806
Malonylation	8736	137	14
N-linked Glycosylation	7916	103976	1842
O-linked Glycosylation	6340	1626	3785
Sumoylation	5450	9943	178
S-nitrosylation	4203	473	324
Glutathionylation	4161	44	92
Amidation	2907	1296	896
Palmitoylation	1094	6766	382
Hydroxylation	1725	2784	285
Pyrrolidone carboxylic acid	908	712	529
Glutarylation	767	45	3
Gamma-carboxyglutamic acid	439	748	87
Crotonylation	368	268	6
Oxidation	359	336	24
Myristoylation	279	1428	182
C-linked Glycosylation	255	104	17
Sulfation	251	1128	120
Formylation	250	33	40
Citrullination	122	351	19
GPI-anchor	82	972	47
Nitration	77	248	15
S-diacylglycerol	57	2073	48
Carboxylation	40	1505	38
Lipoylation	35	566	29
Carbamidation	22	0	1
Neddylation	11	0	4
Pyruvate	9	1636	6
S-linked Glycosylation	6	6	5
Pyrrolylation	0	469	0
**Total in dbPTM 2019**	**908917**	**347984**	**92648**

### High-quality benchmark datasets for the comparison of PTM prediction tools

MS/MS-based proteomic technology has been rapidly developed, which has not only increased the number of sites but has also the discovery of more PTM types. Although numerous computational methods (7,35,36,50–60) have been proposed to predict putative PTM sites based on protein sequence, there is a desperate lack of a standard dataset for the evaluation of predictive powers among various PTM prediction tools. Owing to the abundance of PTM substrate sites, this update follows a rigorous process (61) to construct a non-homologous benchmark dataset for each PTM type. First, the window length of 2*n* + 1 (*n* = 10) was utilized to extract sequence fragments that are centered at the modified sites. For each PTM type with sufficient experimental data, the sequence fragments containing substrate sites are regarded as the positive dataset. On the other hand, given a specific PTM type, the 21-mer sequence fragments centering at the same type of residue are regarded as the negative dataset. Then, the duplicated sequence fragments are removed from the positive or negative datasets by using the CD-HIT program (62). The program cd-hit-2d with 40% sequence identity is further adopted to eliminate the negative sequence fragments that have higher similarity than the positive data. The removal of homologous sequences among positive and negative datasets can prevent the possibility of causing false positive or false negative predictions.

Data balancing is an important preparatory process for model construction, since imbalanced positive and negative datasets might lead to a sub-optimal predictive model with skewed classification. For several PTM types that contain large amounts of experimental data, their benchmark datasets should be manufactured with more strict processing standards. Therefore, if the PTM type has more than 10,000 experimental sites, such as acetylation, N-linked glycosylation and ubiquitination, the sequence fragments with similarity higher than 40% were removed from the positive and negative datasets. Notably, certain PTMs are catalyzed by different enzymes according to their substrate site specificities, such as protein kinases for catalyzing phosphorylation of serine, threonine and tyrosine residues. In this update, the phosphorylated substrate sites are categorized into many groups based on their catalytic kinases, including CDK, MAPK, PKA, PKC, CK2, CAMKL, GSK, AKT, CAMK2, CK1, RSK, GRK, PKG, DYRK, MAPKAPK, DMPK, PKD, PDK1, SGK, RAD53, DAPK, PKN, CAMK1, MLCK and NDR. Numbers of phosphorylated proteins, positive sites and negative sites for each kinase group are presented in Supplementary Table S3. Other PTM types, such as acetylation, carbamidation, citrullination, C-linked glycosylation, crotonylation, formylation, gamma-carboxyglutamic acid, glutarylation, glutathionylation, hydroxylation, lipoylation, malonylation, methylation, nitration, N-linked glycosylation, O-linked glycosylation, phosphorylation, S-diacylglycerol, S-nitrosylation, succinylation, sumoylation and ubiquitination, are also provided.

### Easy-to-use resource portal for PTM analyses

An extensive PTM analysis resource portal has been established to support those looking for appropriate databases or tools to analyze a single or multiple PTMs of interest. In this update, ∼270 online PTM-related databases and tools have been integrated into the resource portal. It is convenient to access the most relevant resources via a newly developed easy-to-use web interface in dbPTM. With the integrative resource portal, users who are interested in PTM analyses do not need to spend much effort to either searching out online resources by using a variety of keywords or manually curating research articles from a large-scale literature database. As depicted in Supplementary Figure S3, users can choose a specific PTM type and then a list of all related databases and tools is displayed with resource name, description, website link and the supporting literature. The integrated resources can provide a variety of functional analyses, including annotation of PTM substrate sites, detection of substrate site motifs, prediction of various PTM sites, annotation of disease association, FEA, structural analysis of substrate sites and construction of regulatory networks, for example. The expanded resource portal should provide users with effective and efficient access to all PTM-related online resources.

### Disease-associated PTM sites explored from SAPs based on GWAS

**Figure 2.**
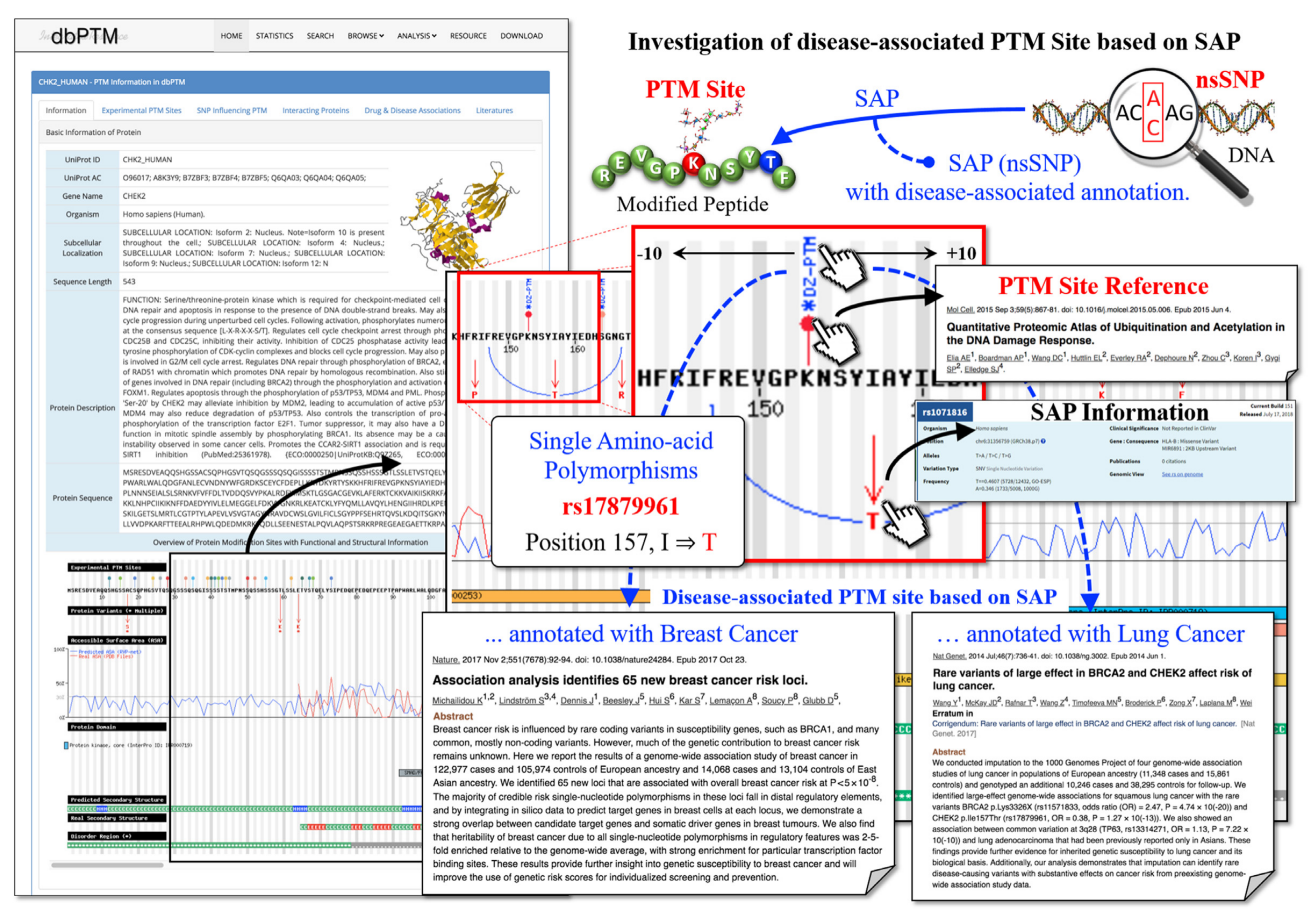
A case study of disease-associated acetylation site (Lys153) on Serine/threonine-protein kinase Chk2 based on the surrounding amino acid variants derived from disease-associated nsSNPs

After a large-scale screening of PTM substrate sites and SAPs, the dbPTM has identified 350 disease-associated PTM sites based on the disease association of GWAS. The number of disease-associated PTM sites for each PTM type and the relevant disease traits are given in Supplementary Table S5. Based on the annotation of disease traits in GWAS, protein phosphorylation has the most abundant data (214 substrate sites) associated with the disease traits, such as immature fraction of reticulocytes, coronary artery disease, high light scatter reticulocyte count, intraocular pressure, high light scatter reticulocyte percentage of red cells, mean corpuscular volume, blood protein levels, body mass index, fibrinogen levels and platelet count. Figure 2 presents a case study of disease-associated lysine acetylation site (Lys153) on serine/threonine-protein kinase Chk2 (CHEK2) based on two neighboring amino acid variants derived from disease-associated SAPs. The two SAPs with dbSNP ID rs17879961 and rs587781667 induce the amino acid variants by changing Ile157 to Thr157 and Arg145 to Pro145, respectively. Detailed information of SAPs can be obtained by clicking on each SAP icon in the visualized protein sequence. It is worth noting that SNP rs17879961 is associated with the progression of various diseases; Michailidou *et al.* have reported that rs17879961 has a statistically significant association with breast cancer risk based on GWAS in European and East Asian ancestry (63). Additionally, Wang *et al.* identified large-effect genome-wide associations for squamous lung cancer with amino acid variants on CHEK2 (Ile157Thr, rs17879961) and BRCA2 (Lys3326X, rs11571833) (64). Due to the linear relationship between the acetylation site (Lys153) and SAP rs17879961 (Ile157Thr) on CHEK2, the acetylation site was identified as having the potential to be disease-associated PTM sites from SAP based on GWAS (41). On dbPTM, the ‘Disease-associated PTM Sites based on SAP’ provides a list of disease-associated PTM sites with the annotations of modified location, PTM type, SAP position, residue variant, dbSNP ID, related diseases and supporting references. Supplementary Figure S4 showcases an efficient scheme for users to access all of the disease-associated PTM sites based on SAP analysis in dbPTM.

### Crosstalk between O-linked glycosylation and phosphorylation

**Figure 3.**
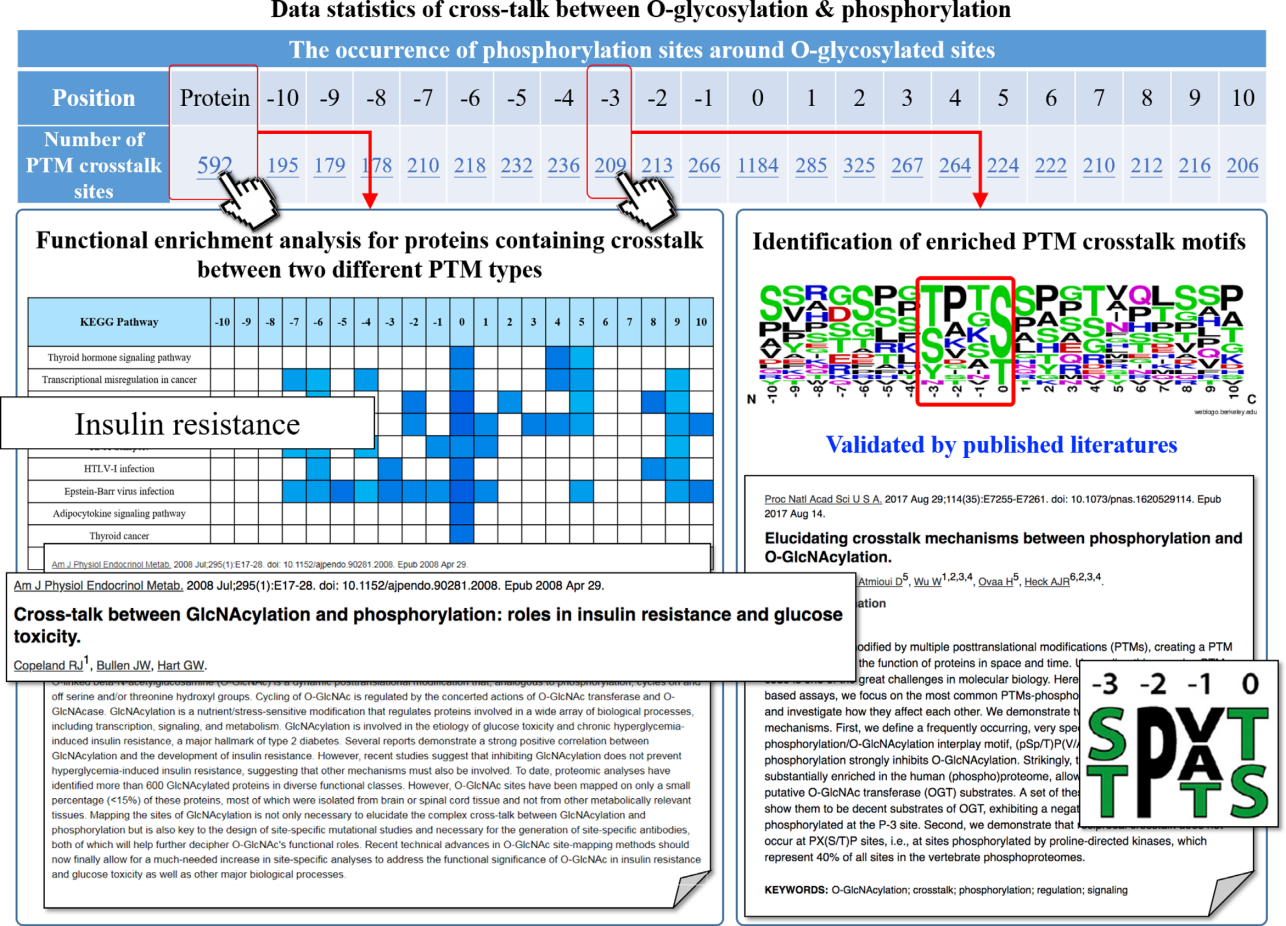
A case study for investigating into the crosstalk between O-glycosylation and phosphorylation.

O-linked glycosylation and phosphorylation are dynamic and inducible PTMs occurring on serine and threonine residues. Concurrent occurrences of phosphorylation and O-glycosylation on the same protein can cross-talk with each other to modulate protein function or activity in several cases (26,27,65). In recent years, increasing interest in understanding the interplay between different PTMs has inspired us to design a platform specifically for investigating the relative frequency and functional relevance of PTM co-occurrences on modified proteins. Figure 3 presents a case study for investigating the co-occurrence of O-glycosylated and phosphorylated substrate sites. In this case, O-glycosylation is regarded as the target PTM, and a table presents all other PTM types co-occurring within a specified distance (-10 ∼ +10 AA) to the O-glycosylation sites. In the table, the columns represent the number of other PTM sites in relative positions relative to the O-glycosylated sites. As shown in the case study, there are 209 phosphorylation sites occurring at position −3 corresponding to the O-glycosylation sites at position 0. This investigation identifies enriched PTM crosstalk motifs between O-glycosylation and phosphorylation, (pS/pT)P(V/A/T)(gS/gT), which has been reported as being substantially enriched in the human phosphoproteome as well as being decent substrates of O-GlcNAc transferase (OGT), and further indicates that phosphorylation strongly inhibits O-GlcNAcylation (66). Additionally, there are 1,184 phosphorylation sites occurring at position 0 that are also O-glycosylated substrate sites. The occurrences of O-glycosylation and phosphorylation at the same position indicate the two PTMs might compete for the same substrate sites of proteins.

To further understand the molecular mechanisms, FEA was performed for all modified proteins that contain a co-occurrence of O-glycosylation and phosphorylation sites. The result indicates that the functional roles of crosstalk between O-glutarylation and phosphorylation is mainly involved in the thyroid hormone signaling pathway, transcriptional misregulation in cancer, insulin resistance and proteoglycans in cancer. A couple of studies have demonstrated a positive correlation between O-glycosylation/phosphorylation and the development of insulin resistance (67–72). GlcNAcylation is abundant on signaling proteins, including those in the insulin signaling pathway and has a complex interplay with phosphorylation. One likely mechanism for HBP-dependent insulin resistance is that the increased GlcNAcylation might antagonize phosphorylation-dependent insulin signaling (73–75). This investigation suggests that the co-occurrence of different PTM sites is important not only for elucidating complex PTM interplay but also for designing site-specific antibodies, both of which will help further deciphering the functional roles of PTM crosstalk.

### Enhanced web interface for functional investigation of PTM sites

**Figure 4.**
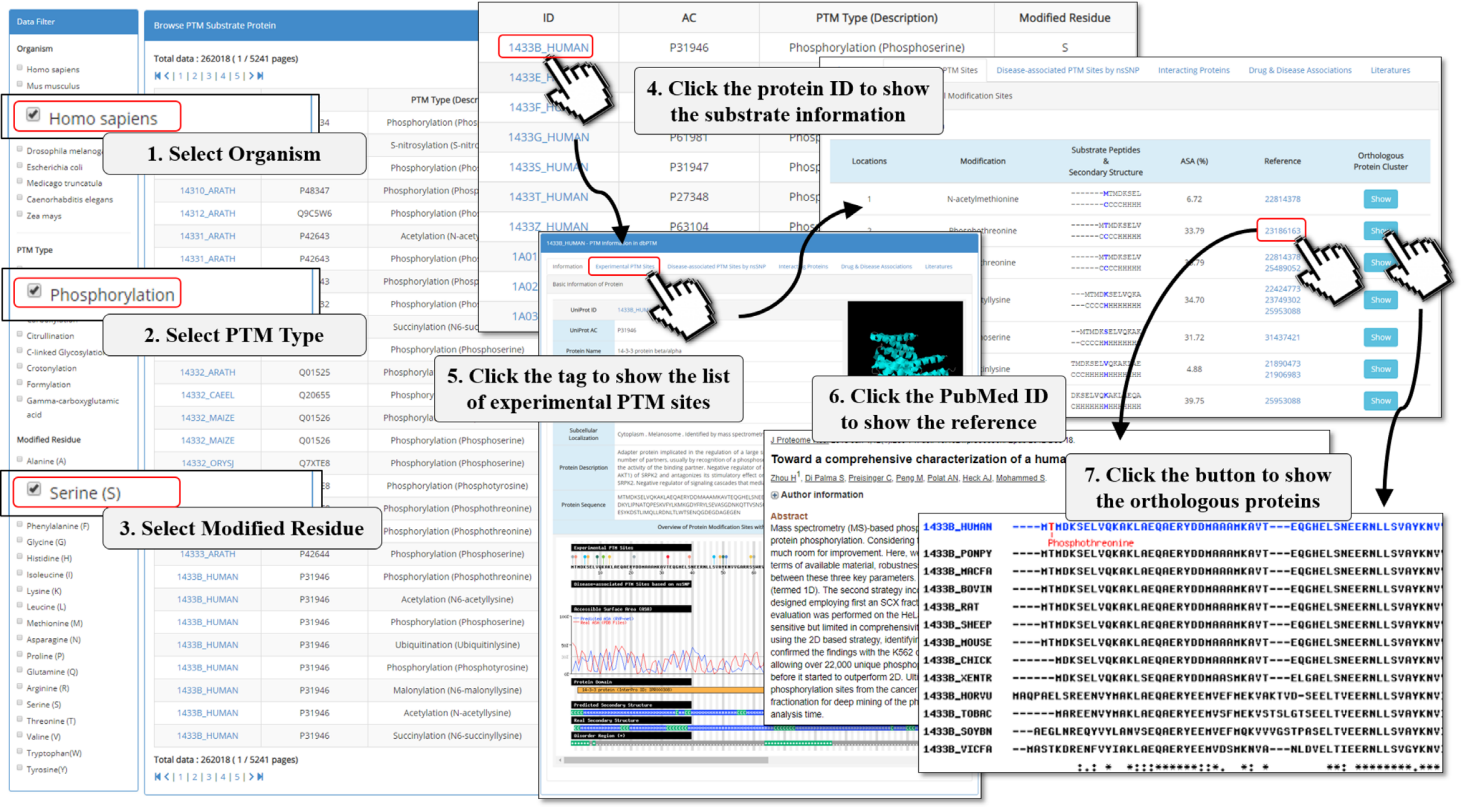
A tutorial for accessing the modified proteins by using the advanced filter functions.

In this update, a more formal scheme was used to redesign the user interface and to enhance the use of dbPTM. Multiple ways can be used to search for the proteins with specific PTMs in this update. Figure 4 shows an example of using the advanced filter function to efficiently find out the PTM substrates that meet selection criteria. Additionally, the information page of each substrate protein has also been improved, as well as the relevant functional and structural information of PTM substrate sites. Moreover, the two newly developed functions ‘Exploring disease-associated PTMs based on SAP’ and ‘Investigation of PTM crosstalk between two different types’ are embedded in the dbPTM website. According to a tidy and easy-to-use design concept, an interactive platform has been developed to efficiently accessing the large scale PTM data. As per the example presented in Supplementary Figure S5, the substrate site specificity of acetylated methionine can be explored through a summary table, including information about modified chemical structure, substrate motif, solvent accessibility, secondary structure, positional amino acid composition and the distribution of subcellular localizations of substrate proteins.

## DISCUSSIONS AND CONCLUSION

Since dbPTM was established in 2006, the database content has been continuously updated. One of the aims of dbPTM is to provide comprehensive information about PTMs with experimentally confirmed evidence. Up till now, the dbPTM has curated ∼92,000 research articles regarding site-specific peptides of various PTMs. As a result, over 900,000 PTM sites have been integrated into dbPTM. Owing to the high-throughput of MS/MS-based technology, large-scale PTM peptides can be identified once in a proteomic experiment. Therefore, an increasing number of PTM-related databases and tools have been developed. With the dramatic increase of online PTM resources, dbPTM has been dedicated to the development of a resource portal to integrate as many as possible online resources for PTM analyses. In this update, ∼270 online databases and tools have been integrated into the resource portal. The expanded resource portal aims to provide users effective and efficient access to all PTM-related online resources. In the analysis of linear relationship between SAPs and PTM sites, this update has revealed more than 168,000 PTM sites that are related to SAPs in 17,694 human proteins. We further identify 350 disease-associated PTM sites based on 1,663 SAPs of GWAS. To understand the possible mechanism underlying PTM crosstalk, 169 PTM pairs have been subjected to motif discovery and FEA based on the co-occurrence of each PTM pair.

In this update, the web interface has been redesigned with a formal scheme to support users accessing functional and structural information of PTMs efficiently and effectively. Users can obtain all the experimental data of various PTM types through the updated download page. The dbPTM will be enhanced continuously by manually curating more literature, recruiting more curators and integrating more databases and tools. In summary, we plan to put great effort for providing truly valuable contributions to the PTM community in terms of the high usage rate of dbPTM.

## DATA AVAILABILITY

The data content in dbPTM will be maintained and updated quarterly by continuously surveying the public resources and research articles. The updated resource is now freely accessed online at http://dbPTM.mbc.nctu.edu.tw/. All of the experimentally verified PTM sites as well as the benchmark dataset could be downloaded in the text format. Additionally, the Supplementary Figures S1–5 and Tables S1–5 are available at NAR online.

## Supplementary Material

Supplementary Data
