# Supplementary material for: dbPTM in 2019: exploring disease association and cross-talk of post-translational modifications
Source: Nucleic Acids Res. 2018 Nov 10;47(Database issue):D298–308. doi: 10.1093/nar/gky1074 (PMC6323979; doi:10.1093/nar/gky1074)
Supplement: Supplementary Data [file gky1074_supplemental_files.doc]

**SUPPLEMENTARY MATERIALS**

**Supplementary Figures**

**Figure S1. Schematic representation of the improvements and advances in dbPTM 2019 update.**

**Figure S2. Investigation of PTM crosstalk between two different types and their functional enrichment analysis.**

**Figure S3. A tutorial for querying the online resources of interested PTM type.**

**Figure S4. A tutorial for querying the disease-associated PTM sites based on SAPs.**

**Figure S5. A tutorial for studying the substrate site specificity of a specific PTM through a summary table.**

**Supplementary Tables**

**Table S1. Comparison of data statistics of experimental and putative PTM sites between dbPTM 2019 and other PTM databases.**

**Table S2. Comparison of data statistics of relevant information between dbPTM 2019 and previous version.**

**Table S3. Data statistics of benchmark dataset for PTM types with sufficient data.**

**Table S4. Summarized table of all integrated tools and databases associated with PTM analyses.**

**Table S5. Distribution of disease or traits for 11 representative PTM types.**


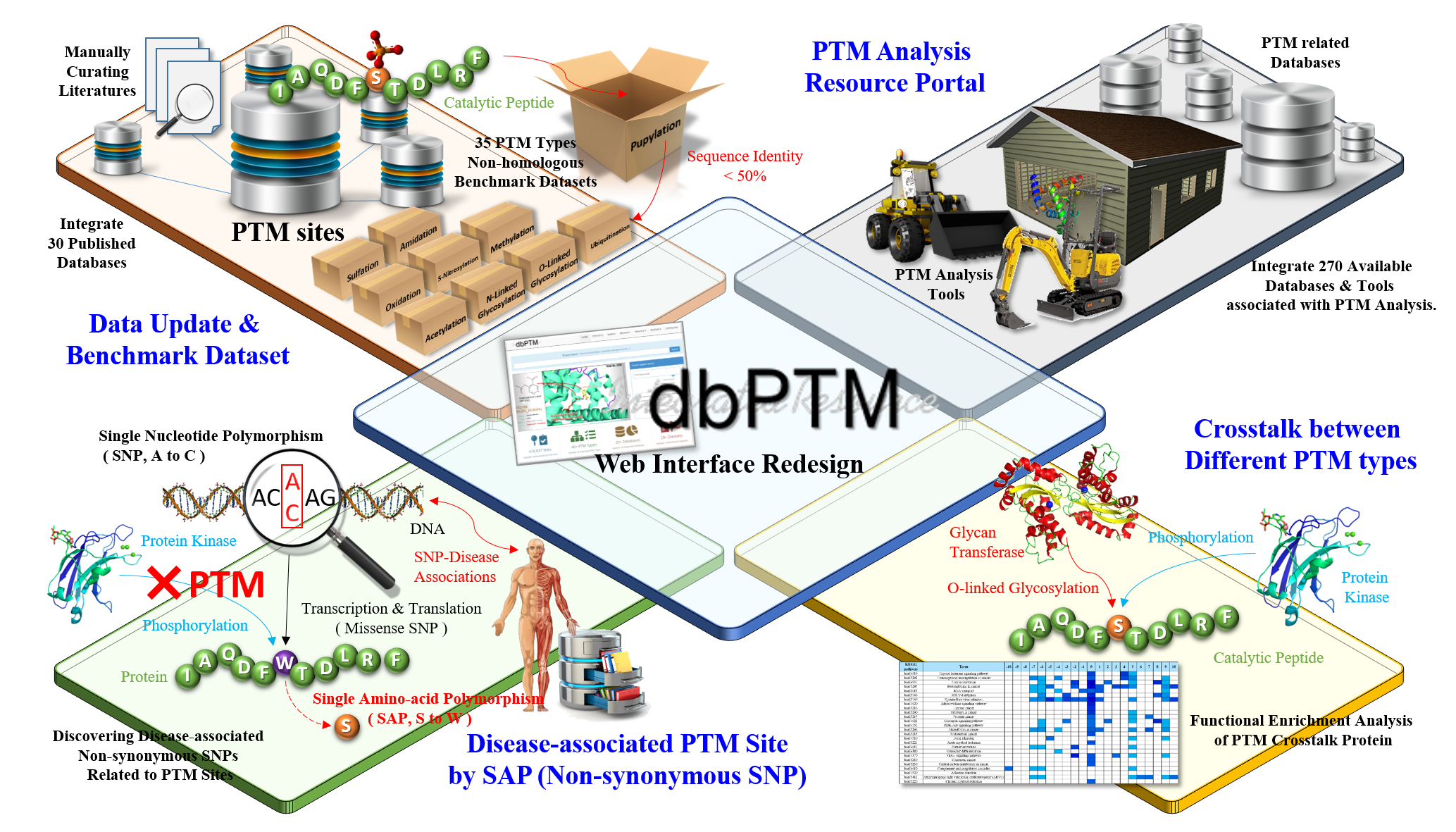


**Figure S1. Schematic representation of the improvements and advances in dbPTM 2019 update.**

**
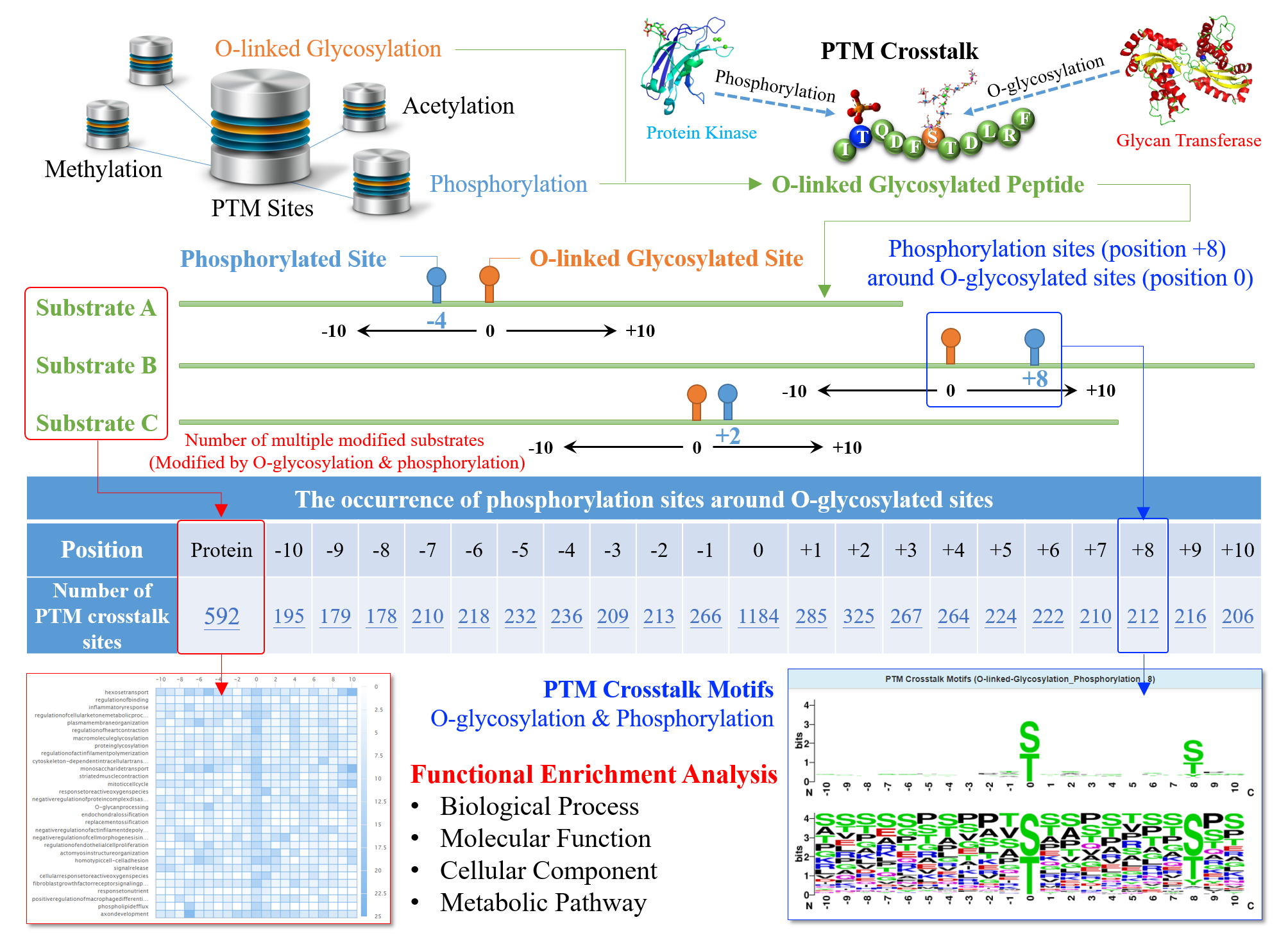
**

**Figure S2. Investigation of PTM crosstalk between two different types and their functional enrichment analysis.**

**
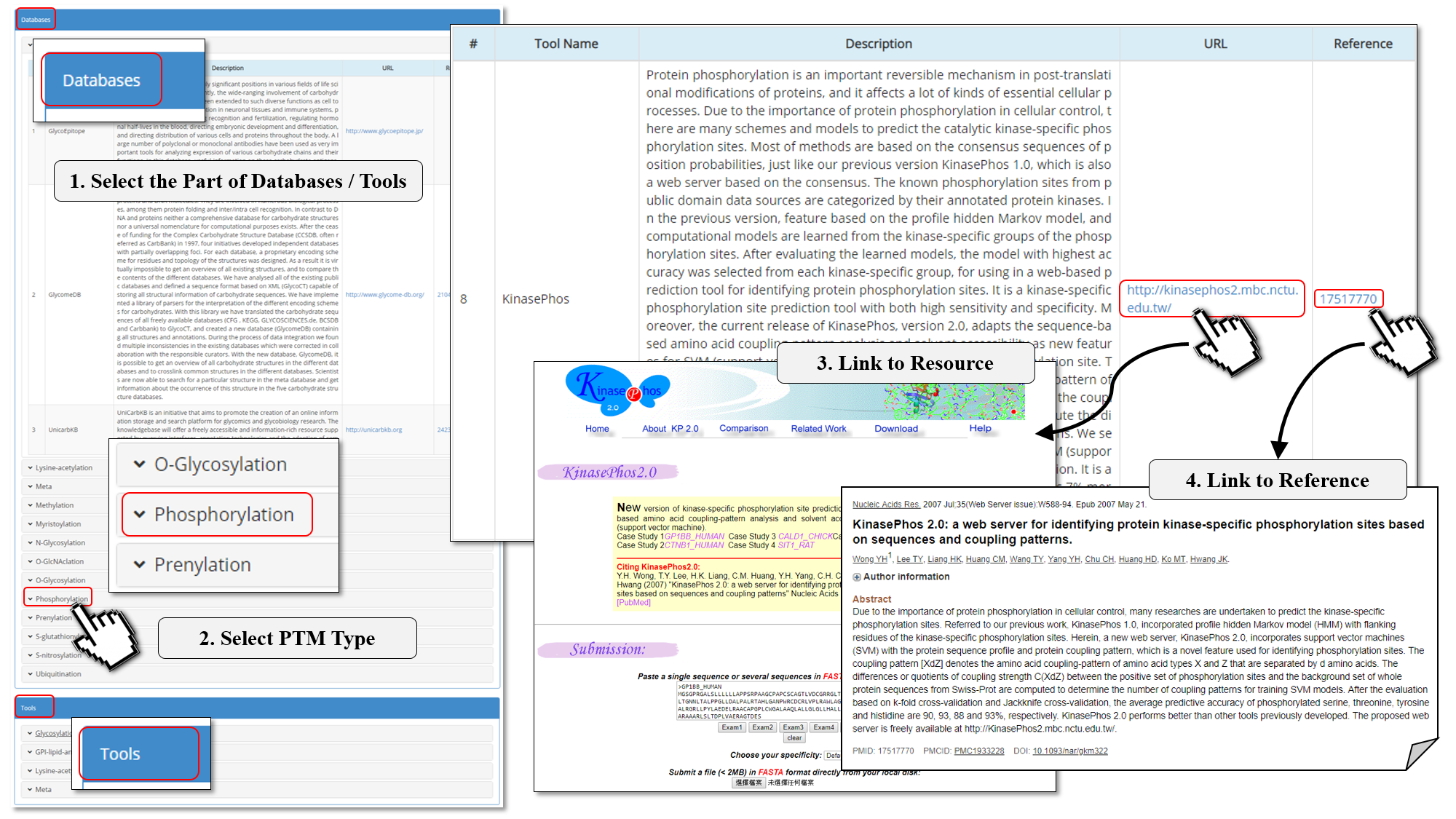
**

**Figure S3. A tutorial for querying the online resources of interested PTM type.** The resource portal has integrated a number of online resources for PTM analysis. These resources are divided into two major categories: Databases and Tools. Users could select a PTM type of interest, a list of resource name, description, website link, and relating references are provided to users. The redesigned resource portal can let users browse easily and access efficiently to the appropriate resources by clicking on the website link to connect to them.


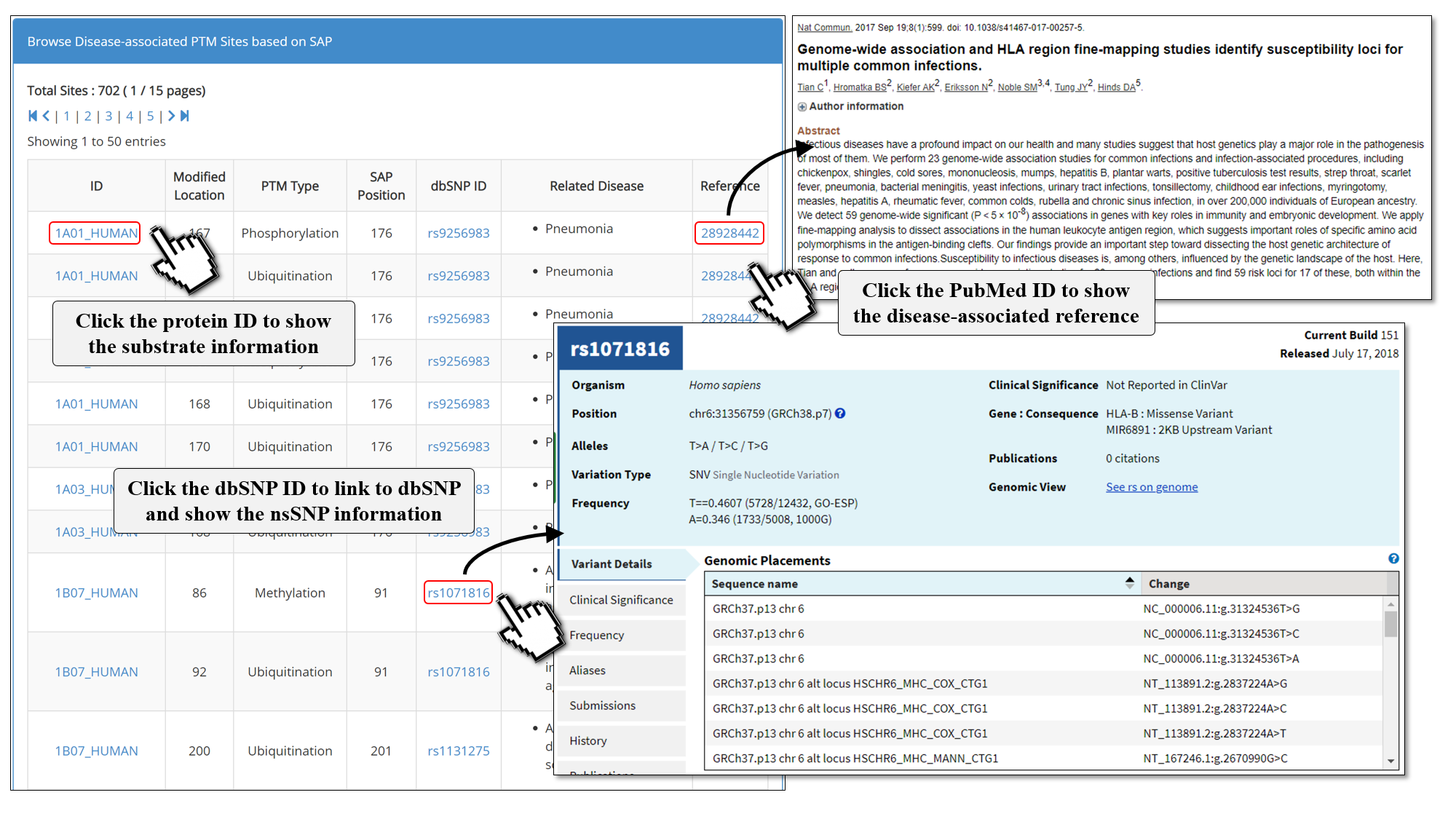


**Figure S4. A tutorial for querying the disease-associated PTM sites based on SAPs.** All of the disease-associated PTM sites based on SAPs have been listed systematically and exclusive represented in a summary table. Users can query the disease-associated PTM sites via the ‘Analysis’ item of the navigation menu at the top of web page and click on ‘UniProt ID’ to view the PTM relevant information of the protein. The ‘dbSNP ID’ and ‘PubMed ID’ can let users to view the SAP information and the disease-associated reference, respectively.


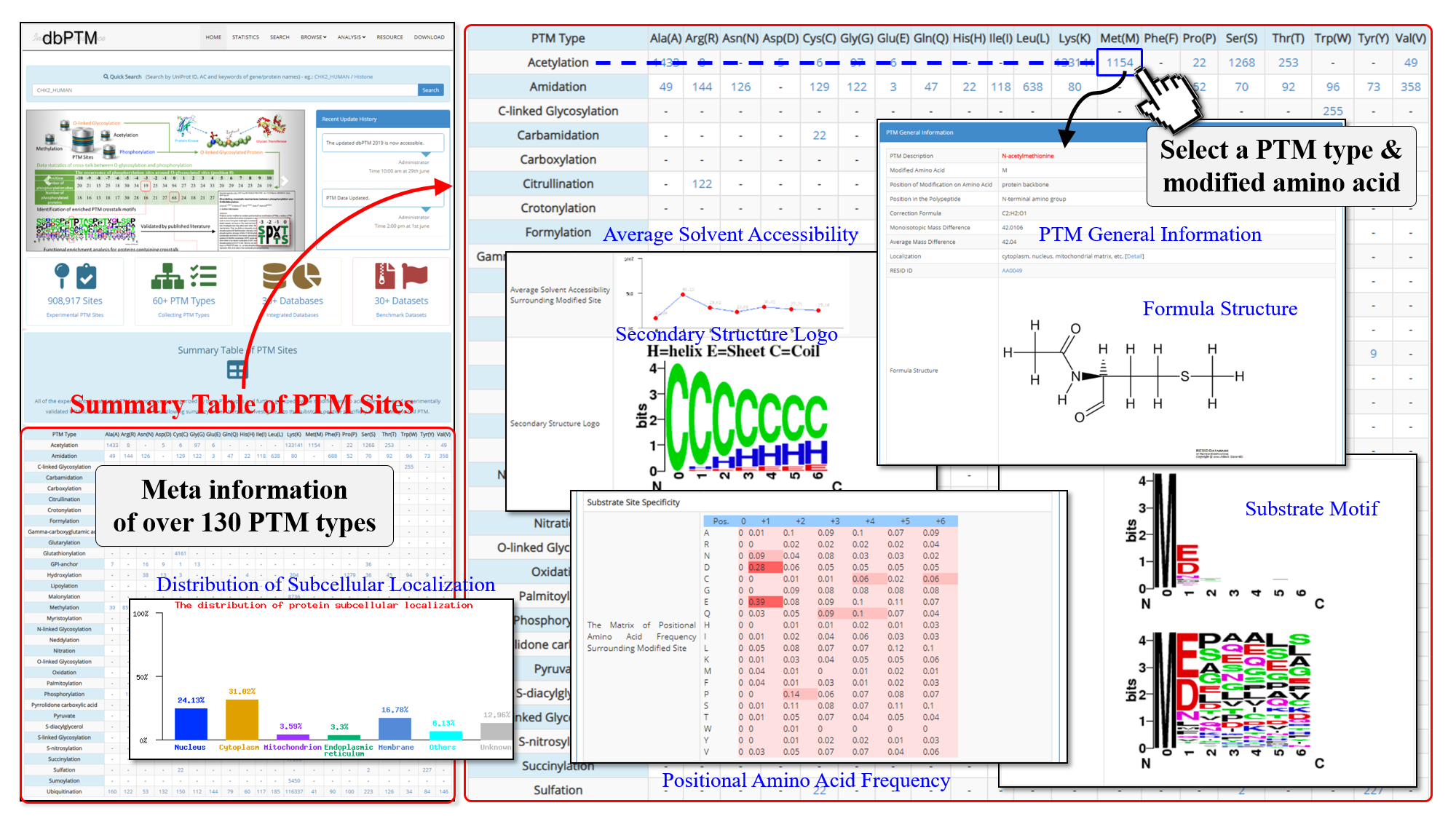


**Figure S5. A tutorial for studying the substrate site specificity of a specific PTM through a summary table.** This update aims to provide users a more convenient way to access the relevant information of over 130 PTM types based on a well-organized summary table, including PTM general information, modified chemical structure, substrate motif, average solvent accessibility, secondary structure, positional amino acid composition, and the distribution of subcellular localization of substrate proteins.

**Table S**1. Comparison of data statistics of experimental and putative PTM sites between dbPTM 2019 and other PTM databases.

| **Resource** | **Version** | **Number of experimental PTM sites** | **Number of putative PTM sites** | **Number of curated research articles** |
| --- | --- | --- | --- | --- |
| ProteomeScout | Release 2018_05 | 15,627 | 688,760 | 40 |
| SysPTM | Release 2.0 | 41,340 | 473,426 | 1,352 |
| dbPAF | Release 1.0 | 483,001 | 0 | 15,139 |
| PhosphoSitePlus | Release 2018_05 | 85,136 | 343,340 | 79,935 |
| ActiveDriverDB | Release 1.0 | 368,005 | 17,181 | 3,682 |
| UniProtKB | Release 2018_03 | 97,172 | 276,643 | 10,080 |
| CPLM | Release 2.0 | 0 | 203,972 | 0 |
| PLMD | Release 3.0 | 184,417 | 362 | 1,590 |
| SubPhosDB | Release 2013_07 | 0 | 137,153 | 0 |
| mUbiSiDa | Release 2013_10 | 111,208 | 0 | 102 |
| HPRD | Release 9.0 | 49,811 | 43,899 | 1,832 |
| Ubisite | Release 1.0 | 91,588 | 844 | 24,988 |
| PHOSIDA | Release 3.69 | 80,062 | 0 | 17 |
| dbPPT | Release 1.0 | 69,297 | 0 | 79 |
| Phospho.ELM | Release 9.0 | 50,622 | 38 | 3,589 |
| UbiNet | Release 1.0 | 44,175 | 0 | 233 |
| PhosphoGRID | Release 2.0 | 42,908 | 0 | 528 |
| ESBL | Release 2018_05 | 9,970 | 10,031 | 5 |
| dbPSP | Release 1.0 | 0 | 7,391 | 0 |
| SwissPalm | Release 2.0 | 5,384 | 0 | 425 |
| ASEB | Release 2015_12 | 4,875 | 0 | 281 |
| CarbonylDB | Release 2018_05 | 4,198 | 13 | 112 |
| dbSNO | Release 2.0 | 4,161 | 4 | 175 |
| dbGSH | Release 1.0 | 4,119 | 0 | 60 |
| OGlycBase | Release 6.0 | 2,211 | 10 | 178 |
| ProteomeXchange | Release 2018_05 | 464 | 75 | 243 |
| Mal-Lys | Release 1.0 | 0 | 483 | 0 |
| MeMo | Release 2.0 | 364 | 0 | 57 |
| PupDB | Release 1.3 | 195 | 116 | 4 |
| PredGPI | Release 1.0 | 0 | 26 | 0 |
| **dbPTM** | **Release 2019** | **908,917** | **347,984** | **92,648** |

**Table S2. Comparison of data statistics of relevant information between dbPTM 2019 and previous version.**

| **Description** | **Previous version** | **dbPTM 2019** |
| --- | --- | --- |
| Experimental validated PTM sites | 610,037 | 908,917 |
| Protein general information | 96,595 | 128,003 |
| Protein variant | 84,263 | 25,257,297 |
| Protein sequence | 536,789 | 557,713 |
| Secondary structure information | 535,981 | 557,676 |
| Tertiary structure information | 38,253 | 166,743 |
| Accessible surface area | 536,789 | 557,713 |
| Disorder region | 332,490 | 557,713 |
| Protein-protein interaction | 121,306 | 1,573,731 |
| Integrated online resource | 148 | 270 |
| Benchmark dataset | 13 | 30 |
| Disease-associated PTM sites | N/A | 350 |
| PTM pairs (PTM crosstalk) | N/A | 169 |

**Table S3. Data statistics of benchmark dataset for PTM types with sufficient data.**

| **PTM Type** | **Number of proteins** | **Number of positive sites** | **Number of negative sites** |
| --- | --- | --- | --- |
| Acetylation | 10074 | 14407 | 8664 |
| Carbamidation | 19 | 20 | 411 |
| Citrullination | 67 | 76 | 1501 |
| C-linked Glycosylation | 22 | 197 | 331 |
| Crotonylation | 69 | 117 | 36 |
| Formylation | 159 | 172 | 1452 |
| Gamma-carboxyglutamic acid | 110 | 319 | 553 |
| Glutarylation | 210 | 725 | 2543 |
| Glutathionylation | 2337 | 3555 | 6617 |
| Hydroxylation | 385 | 1273 | 2876 |
| Lipoylation | 28 | 29 | 779 |
| Malonylation | 3319 | 8119 | 16625 |
| Methylation | 7017 | 14686 | 36502 |
| Nitration | 61 | 66 | 982 |
| N-linked Glycosylation | 5972 | 8859 | 10199 |
| O-linked Glycosylation | 1345 | 5249 | 50373 |
| Phosphorylation by AKT | 351 | 380 | 14617 |
| Phosphorylation by CAMK1 | 34 | 44 | 2342 |
| Phosphorylation by CAMK2 | 254 | 366 | 12575 |
| Phosphorylation by CAMKL | 454 | 556 | 20129 |
| Phosphorylation by CDK | 1020 | 1503 | 29823 |
| Phosphorylation by CK1 | 174 | 339 | 5808 |
| Phosphorylation by CK2 | 511 | 819 | 15387 |
| Phosphorylation by DAPK | 51 | 53 | 1284 |
| Phosphorylation by DMPK | 99 | 109 | 3533 |
| Phosphorylation by DYRK | 109 | 142 | 3470 |
| Phosphorylation by GRK | 77 | 147 | 2310 |
| Phosphorylation by GSK | 291 | 397 | 10328 |
| Phosphorylation by MAPK | 857 | 1270 | 22436 |
| Phosphorylation by MAPKAPK | 100 | 125 | 3096 |
| Phosphorylation by MLCK | 20 | 34 | 484 |
| Phosphorylation by NDR | 28 | 32 | 1096 |
| Phosphorylation by PDK1 | 77 | 93 | 2274 |
| Phosphorylation by PKA | 905 | 1209 | 29813 |
| Phosphorylation by PKC | 691 | 943 | 24207 |
| Phosphorylation by PKD | 88 | 97 | 3401 |
| Phosphorylation by PKG | 126 | 145 | 7311 |
| Phosphorylation by PKN | 26 | 50 | 866 |
| Phosphorylation by RAD53 | 29 | 75 | 1560 |
| Phosphorylation by RSK | 221 | 215 | 6985 |
| Phosphorylation by SGK | 63 | 77 | 3057 |
| S-diacylglycerol | 57 | 57 | 59 |
| S-nitrosylation | 2302 | 3592 | 5803 |
| Succinylation | 3100 | 5064 | 5499 |
| Sumoylation | 179 | 9590 | 130649 |
| Ubiquitination | 5958 | 10437 | 8143 |

**Table S4**. Summarized table of all integrated tools and databases associated with PTM analyses.

| **PTM type** | **Number of Integrated tools** | **Tool name** | **Number of integrated databases** | **Database name** |
| --- | --- | --- | --- | --- |
| Acetylation | 10 | Acetylation Set | 1 | Writers Erasers |
|  |  | EnsemblePail |  |  |
|  |  | Extraction and C |  |  |
|  |  | GPS-PAIL |  |  |
|  |  | lysine (K) Acety |  |  |
|  |  | N-Terminal Acety |  |  |
|  |  | NetAcet |  |  |
|  |  | PLMLA |  |  |
|  |  | Prediction of Ac |  |  |
|  |  | PSKAcePred |  |  |
| Amidation | 1 | Prediction of Am | 0 |  |
| Carbonylation | 4 | CarSPred | 1 | CarbonylDB |
|  |  | iCar-PseCp |  |  |
|  |  | predCar-site |  |  |
|  |  | Carboxylator |  |  |
| Crosstalk | 2 | ModPred | 2 | CrosstalkDB |
|  |  | PTM cross-talk - |  | PTMCode |
| DePhosphorylation | 1 | DephosSite | 0 |  |
| Formylation | 1 | NformPred | 0 |  |
| Glutathione | 2 | GSHSite | 0 |  |
|  |  | GSTPred |  |  |
| Glycation | 3 | Gly-PseAAC | 0 |  |
|  |  | Identifying Prot |  |  |
|  |  | NetGlycate |  |  |
| Glycosylation | 13 | DictyOGlyc | 12 | Aglgenes  E. coli K antige  GlycoBase  GlycoFish  GlycoFly  GlycomeAtlas  GlycoProtDB – GP  GlycoSuiteDB  Japan Consortium  O-GlycBase  ProGlycProt  UniPep |
|  |  | GlycoDomain View |  |  |
|  |  | GLYCOsites Predi |  |  |
|  |  | Glycosylation Pr |  |  |
|  |  | Glypre |  |  |
|  |  | GlyProt |  |  |
|  |  | GlySeq |  |  |
|  |  | Integrated Glyco |  |  |
|  |  | N-linked Glycosy |  |  |
|  |  | NetNGlyc |  |  |
|  |  | NetOGlyc |  |  |
|  |  | O-GlcNAcPRED |  |  |
|  |  | Plant Lectin Dom |  |  |
| Gpi-anchor | 4 | big-Pi plant | 0 |  |
|  |  | big-Pi predictor |  |  |
|  |  | GPI-SOM |  |  |
|  |  | PredGPI |  |  |
| Hydroxylation | 4 | identify Hydroxy | 0 |  |
|  |  | iHyd-PseCp |  |  |
|  |  | PredHydroxy |  |  |
|  |  | RF-Hydroxysite |  |  |
| Malonylation | 2 | Malonylation Lys | 0 |  |
|  |  | MaloPred |  |  |
| Mannosylation | 1 | NetCGlyc | 0 |  |
| Meta | 5 | CWM Global Searc motif extractor  Post-translation PTMOracle SysPTM | 9 | Biochemical Subs |
|  |  |  |  | iPTMnet |
|  |  |  |  | Mining Protein P |
|  |  |  |  | MsViz |
|  |  |  |  | Post Translation |
|  |  |  |  | ProteomeScout |
|  |  |  |  | PTMCurator |
|  |  |  |  | Terminus Oriente |
|  |  |  |  | topPTM |
| Methylation | 9 | GPS-Methyl-group | 0 |  |
|  |  | iLM-2L |  |  |
|  |  | iMethyl-PseAAC |  |  |
|  |  | MePred-RF |  |  |
|  |  | MethK |  |  |
|  |  | MethylQuant |  |  |
|  |  | PMes |  |  |
|  |  | Prediction of Sp |  |  |
|  |  | Protein aRginine |  |  |
| N-myristoylation | 4 | GPS-LIPID | 0 |  |
|  |  | Myristoylator |  |  |
|  |  | NMT |  |  |
|  |  | Plant-Specific M |  |  |
| Oxidative | 0 |  | 1 | RedoxDB |
| Palmitoylation | 5 | CKSAAP-Palm | 1 | SwissPalm |
|  |  | Clustering and S |  |  |
|  |  | NBA-Palm |  |  |
|  |  | SeqPalm |  |  |
|  |  | WAP-Palm |  |  |
| Phosphoglycerylation | 3 | CKSAAP_PhoglySit | 0 |  |
|  |  | iPGK-PseAAC |  |  |
|  |  | Phogly-PseAAC |  |  |
| Phosphorylation | 49 | ArMone | 24 | ActiveDriverDB Collecting Duct  database of Phos dbPSP Human Protein Re Kinase-Associate KiPho LymPHOS PepBase PHOSIDA Phospho.ELM Phospho3D PhosphoGRID PhosphoNET PhosphoNetworks PhosphoSitePlus  PhospoPep PhosSNP Plant Protein Ph PTMfunc RIKEN Plant Phos scan-x Soybean Knowledg SubPhosDB |
|  |  | AutoMotif Servic |  |  |
|  |  | CRPhos |  |  |
|  |  | DAPPLE |  |  |
|  |  | DISorder-enhance |  |  |
|  |  | GPS-Polo |  |  |
|  |  | Group-based Pred |  |  |
|  |  | Inference of Kin |  |  |
|  |  | iPhos-PseEn |  |  |
|  |  | KinasePhos |  |  |
|  |  | Kinome Viewer - |  |  |
|  |  | KinomeXplorer |  |  |
|  |  | Maximal Motif Fi |  |  |
|  |  | MetaPredPS |  |  |
|  |  | Motif Descriptio |  |  |
|  |  | MusiteDeep |  |  |
|  |  | Mutation IMpact |  |  |
|  |  | NetPhorest |  |  |
|  |  | NetPhos |  |  |
|  |  | NetPhosBac |  |  |
|  |  | NetPhosK |  |  |
|  |  | NetPhosYeast |  |  |
|  |  | Phos3D |  |  |
|  |  | PhoScan |  |  |
|  |  | PhosContext2vec |  |  |
|  |  | PhosPhAt |  |  |
|  |  | PhosphoPath |  |  |
|  |  | PhosphoPICK |  |  |
|  |  | PhosphoPICK-SNP |  |  |
|  |  | PhosphoRice |  |  |
|  |  | PhosphOrtholog |  |  |
|  |  | Phosphorylation |  |  |
|  |  | PHOsphosite-XTRa |  |  |
|  |  | PhosphoSVM |  |  |
|  |  | PhosPred-RF |  |  |
|  |  | phos_pred |  |  |
|  |  | pkaPS |  |  |
|  |  | PKIS |  |  |
|  |  | PlantPhos |  |  |
|  |  | Prediction of PK |  |  |
|  |  | prediction of Po |  |  |
|  |  | Predikin |  |  |
|  |  | PTMProber |  |  |
|  |  | PyTMs |  |  |
|  |  | Rice_Phospho |  |  |
|  |  | Rule-based Liter |  |  |
|  |  | SubPhos |  |  |
|  |  | TyrPred |  |  |
|  |  | ViralPhos |  |  |
| Prenylation | 2 | iPreny-PseAAC | 1 | PRENbase |
|  |  | Prenylation Pred |  |  |
| Propionylation | 1 | ProSeek | 0 |  |
| Pupylation | 6 | GPS-PUP | 1 | PupDB |
|  |  | IMP–PUP |  |  |
|  |  | iPUP |  |  |
|  |  | profile-based PU |  |  |
|  |  | PU learning for |  |  |
|  |  | Pupylation Predi |  |  |
| S-glutathionylation | 0 |  | 1 | dbGSH |
| S-nitrosylation | 5 | GPS-SNO | 3 | dbPTM  dbSNO  S-Nitros(yl)atio |
|  |  | iSNO-AAPair |  |  |
|  |  | iSNO-PseAAC |  |  |
|  |  | iSulf-Cys |  |  |
|  |  | MDD-SOH |  |  |
| Succinylation | 9 | iPTM-mLys | 0 |  |
|  |  | iSuc-PseAAC |  |  |
|  |  | iSuc-PseOpt |  |  |
|  |  | Position Specifi |  |  |
|  |  | Predicting lysin |  |  |
|  |  | pSuc-Lys |  |  |
|  |  | SuccFind |  |  |
|  |  | SuccinSite |  |  |
|  |  | Succinylation us |  |  |
| Sumoylation | 5 | GPS-SUMO | 0 |  |
|  |  | Joined Advanced |  |  |
|  |  | pSumo-CD |  |  |
|  |  | SUMOAMVR |  |  |
|  |  | SUMOhydro |  |  |
| Tyrosine-nitration | 5 | GPS-YNO2 | 0 |  |
|  |  | iNitro-Tyr |  |  |
|  |  | GPS-TSP |  |  |
|  |  | Sulfation |  |  |
|  |  | The Sulfinator |  |  |
| Ubiquitination | 7 | ESA-UbiSite | 4 | hUbiquitome  Protein Lysine M  UbiBrowser  UbiNet |
|  |  | hCKSAAP_UbSite |  |  |
|  |  | iUbiq-Lys |  |  |
|  |  | UbiPred |  |  |
|  |  | UbiProber |  |  |
|  |  | UbiSite |  |  |
|  |  | UbPred |  |  |

**Table S5. Distribution of disease or traits** for 11 representative PTM types.

| **PTM type** | **Number of PTM sites** | **Number of SAPs** | **Distribution of traits or diseases** |
| --- | --- | --- | --- |
| Phosphorylation | 213 | 156 | Immature fraction of reticulocytes (16 PTM sites) |
|  |  |  | Coronary artery disease (14 PTM sites) |
|  |  |  | High light scatter reticulocyte count (14 PTM sites) |
|  |  |  | Intraocular pressure (10 PTM sites) |
|  |  |  | GIP levels in response to oral glucose tolerance test (120 minutes) (9 PTM sites) |
|  |  |  | High light scatter reticulocyte percentage of red cells (9 PTM sites) |
|  |  |  | Mean corpuscular volume (9 PTM sites) |
|  |  |  | Blood protein levels (8 PTM sites) |
|  |  |  | Mean platelet volume (8 PTM sites) |
|  |  |  | Body mass index (7 PTM sites) |
|  |  |  | Fibrinogen levels (7 PTM sites) |
|  |  |  | Platelet count (7 PTM sites) |
| Acetylation | 47 | 41 | Fibrinogen levels (6 PTM sites) |
|  |  |  | Alcohol dependence (5 PTM sites) |
|  |  |  | Behavioural disinhibition (generation interaction) (5 PTM sites) |
|  |  |  | Cholesterol, total (5 PTM sites) |
|  |  |  | HDL cholesterol (5 PTM sites) |
|  |  |  | Height (5 PTM sites) |
|  |  |  | Hematocrit (5 PTM sites) |
|  |  |  | C-reactive protein (4 PTM sites) |
|  |  |  | C-reactive protein levels (4 PTM sites) |
|  |  |  | C-reactive protein levels or HDL-cholesterol levels (pleiotropy) (4 PTM sites) |
|  |  |  | C-reactive protein levels or LDL-cholesterol levels (pleiotropy) (4 PTM sites) |
|  |  |  | C-reactive protein levels or total cholesterol levels (pleiotropy) (4 PTM sites) |
|  |  |  | Granulocyte count (4 PTM sites) |
|  |  |  | Myeloid white cell count (4 PTM sites) |
|  |  |  | Neutrophil count (4 PTM sites) |
|  |  |  | Neutrophil percentage of white cells (4 PTM sites) |
|  |  |  | Sum basophil neutrophil counts (4 PTM sites) |
|  |  |  | Sum neutrophil eosinophil counts (4 PTM sites) |
| Ubiquitination | 44 | 67 | Inflammatory bowel disease (5 PTM sites) |
|  |  |  | Pneumonia (5 PTM sites) |
|  |  |  | Schizophrenia (5 PTM sites) |
|  |  |  | Autism spectrum disorder or schizophrenia (4 PTM sites) |
|  |  |  | Alzheimer's disease (cognitive decline) (3 PTM sites) |
|  |  |  | Ulcerative colitis (3 PTM sites) |
|  |  |  | Alzheimer's disease (late onset) (2 PTM sites) |
|  |  |  | Crohn's disease (2 PTM sites) |
|  |  |  | Epstein-Barr virus copy number in lymphoblastoid cell lines (2 PTM sites) |
|  |  |  | Height (2 PTM sites) |
|  |  |  | IgG glycosylation (2 PTM sites) |
|  |  |  | Intelligence (MTAG) (2 PTM sites) |
| N-linked glycosylation | 19 | 18 | Blood protein levels (5 PTM sites) |
|  |  |  | Alcohol consumption (transferrin glycosylation) (1 PTM sites) |
|  |  |  | Asparaginase hypersensitivity in acute lymphoblastic leukemia (1 PTM sites) |
|  |  |  | Creutzfeldt-Jakob disease (1 PTM sites) |
|  |  |  | Hematocrit (1 PTM sites) |
|  |  |  | Intraocular pressure (2 PTM sites) |
|  |  |  | Major depressive disorder (1 PTM sites) |
|  |  |  | Mean corpuscular hemoglobin concentration (1 PTM sites) |
|  |  |  | Mean corpuscular volume (1 PTM sites) |
|  |  |  | Multiple sclerosis (1 PTM sites) |
|  |  |  | Prion diseases (1 PTM sites) |
|  |  |  | Severe malaria (1 PTM sites) |
|  |  |  | Sex hormone-binding globulin levels (1 PTM sites) |
|  |  |  | Systemic lupus erythematosus or rheumatoid arthritis (1 PTM sites) |
|  |  |  | Testosterone levels (1 PTM sites) |
|  |  |  | Total cholesterol levels in HDL (1 PTM sites) |
|  |  |  | Urinary albumin-to-creatinine ratio (1 PTM sites) |
| O-linked glycosylation | 11 | 8 | B-type natriuretic peptide to N-terminal pro B-type natriuretic peptide ratio (5 PTM sites) |
|  |  |  | N-terminal pro B-type natriuretic peptide levels (5 PTM sites) |
|  |  |  | Blood protein levels (2 PTM sites) |
|  |  |  | Lymphocyte counts (2 PTM sites) |
|  |  |  | Alzheimer's disease (late onset) (1 PTM sites) |
|  |  |  | Cardiovascular risk factors (1 PTM sites) |
|  |  |  | Cholesterol, total (1 PTM sites) |
|  |  |  | Coronary artery disease (1 PTM sites) |
|  |  |  | Coronary artery disease (myocardial infarction, percutaneous transluminal coronary angioplasty, coronary artery bypass grafting, angina or chromic ischemic heart disease) (1 PTM sites) |
|  |  |  | HDL cholesterol (1 PTM sites) |
|  |  |  | High light scatter reticulocyte count (1 PTM sites) |
|  |  |  | High light scatter reticulocyte percentage of red cells (1 PTM sites) |
|  |  |  | Ideal cardiovascular health (clinical and behavioural) (1 PTM sites) |
|  |  |  | Immature fraction of reticulocytes (1 PTM sites) |
|  |  |  | LDL cholesterol (1 PTM sites) |
|  |  |  | LDL cholesterol levels (1 PTM sites) |
|  |  |  | Lipid metabolism phenotypes (1 PTM sites) |
|  |  |  | Lipid traits (1 PTM sites) |
|  |  |  | Lipoprotein (a) levels (1 PTM sites) |
|  |  |  | Lipoprotein phospholipase A2 activity in cardiovascular disease (1 PTM sites) |
|  |  |  | Lipoprotein(a) levels adjusted for apolipoprotein(a) isoforms (1 PTM sites) |
|  |  |  | Lipoprotein-associated phospholipase A2 activity change in response to darapladib treatment in cardiovascular disease (1 PTM sites) |
|  |  |  | Low density lipoprotein cholesterol (1 PTM sites) |
|  |  |  | Metabolite levels (lipoprotein measures) (1 PTM sites) |
|  |  |  | Pulse pressure (1 PTM sites) |
|  |  |  | Red cell distribution width (1 PTM sites) |
|  |  |  | Response to statins (LDL cholesterol change) (1 PTM sites) |
|  |  |  | Reticulocyte count (1 PTM sites) |
|  |  |  | Reticulocyte fraction of red cells (1 PTM sites) |
|  |  |  | Total cholesterol levels (1 PTM sites) |
| S-nitrosylation | 5 | 3 | Alcohol consumption (transferrin glycosylation) (2 PTM sites) |
|  |  |  | Chronic obstructive pulmonary disease (2 PTM sites) |
|  |  |  | Mean corpuscular hemoglobin (1 PTM sites) |
|  |  |  | Mean corpuscular hemoglobin concentration (1 PTM sites) |
|  |  |  | Mean corpuscular volume (1 PTM sites) |
|  |  |  | Red cell distribution width (1 PTM sites) |
| Methylation | 4 | 5 | Idiopathic intracranial hypertension (2 PTM sites) |
|  |  |  | Alanine aminotransferase (ALT) levels after remission induction therapy lymphoblastic leukemia (ALL) (1 PTM sites) |
|  |  |  | Anti-thyroid drug induced agranulocytosis (1 PTM sites) |
| Crotonylation | 4 | 3 | Height (4 PTM sites) |
| Malonylation | 1 | 2 | High light scatter reticulocyte count (1 PTM sites) |
|  |  |  | High light scatter reticulocyte percentage of red cells (1 PTM sites) |
|  |  |  | Immature fraction of reticulocytes (1 PTM sites) |
| Citrullination | 1 | 2 | Blood protein levels (1 PTM sites) |
| Sumoylation | 1 | 1 | Asparaginase-induced acute pancreatitis in acute lymphoblastic leukemia (onset time) (1 PTM sites) |
